# Relative bradycardia presented as a clinical feature of *Brucella melitensis* infection: A case report

**DOI:** 10.3389/fmed.2022.1013294

**Published:** 2022-12-07

**Authors:** Kai Huang, Xuxia Yu, Yushan Yu, Yin Cen, Jie Shu, Naibin Yang, Jinguo Chu

**Affiliations:** Ningbo First Hospital, Ningbo, China

**Keywords:** brucellosis, *Brucella melitensis*, relative bradycardia, clinical feature, heart rate and body temperature relationship

## Abstract

Brucellosis, caused by *Brucella* species, is an infectious disease transmitted through contact with infected animals or their secretions. The clinical disease is characterized by fever and headache. Relative bradycardia is an inappropriate response of heart rate to body temperature, in which the heart rate does not increase proportionally despite a high fever. In this report, we document one case of *Brucella melitensis* infection demonstrating relative bradycardia. To our knowledge, this is the first report of relative bradycardia in a patient with brucellosis.

## Introduction

Brucellosis is a zoonotic disease that causes more than 500,000 new infections per year across the world. The disease is usually transmitted from animals to humans through contact with infected animals or by consuming contaminated animal products. Common symptoms of brucellosis include headache, fever, fatigue, sweating, joint pain, and weakness (1, 2). Brucellosis is still very common and often considered a neglected disease (3). Relative bradycardia is an important diagnostic clue in a variety of infectious and non-infectious diseases (4, 5). Previous studies found that relative bradycardia was not associated with brucellosis (4, 5). In this study, we report a case of *Brucella melitensis* infection exhibiting relative bradycardia. To the best of our knowledge, no similar cases have previously been reported in the literature.

## Case presentation

On 24 May 2021, a 51-year-old woman was admitted to our hospital with a 1-week history of fever. She developed a fever of 39.5°C with chills that lasted for about 20–30 min as well as cough, headaches, sweating, and fatigue. The patient was evaluated in an emergency department 5 days previous to the presentation. On examination, blood analysis demonstrated normal white blood cell counts (4.25 × 10^9^/L) and neutrophil percentage (63.7%), and elevated C-reactive protein (CRP, 8.66 mg/L) with chest computed tomography (CT) within normal limits. The patient was treated with ceftriaxone (2g qd ivgtt) for 2 days but body temperatures remained elevated (>39–40°C) and other clinical symptoms also persisted. The patient was admitted for hospitalization.

The patient's history indicated that she was a farmer and worked with sheep. The patient reported no previous history of symptoms and was not on medication that could cause symptoms. Other family members did not report similar symptoms. Physical examination was within normal limits. Laboratory results indicated significant elevations of CRP and serum amyloid protein A (SAA) and moderate to minor elevations of D-dimer, alanine aminotransferase, aspartate aminotransferase, adenosine deaminase, creatinine, procalcitonin (PCT), and hepatitis B surface antibody (Table 1).

**Table 1:** Laboratory examination results of the case at admission.

**Items**	**Values**	**Reference range**
White blood cell count ( × 10^9^ /L)	3.62	3.50–9.50
Neutrophil count ( × 10^9^ /L)	2.4	1.8–6.3
Lymphocyte count ( × 10^9^ /L)	0.9	1.1–3.2
Monocyte count ( × 10^9^ /L)	0.4	0.1–0.6
Red blood cell count ( × 10^12^ /L)	4.32	3.8–5.1
Hemoglobin (g/L)	115	115.0–150.0
Platelet count ( × 10^9^ /L)	134	125.0–350.0
High sensitivity C-reactive protein(mg/l)	48.13	0–5.0
D-dimer(ng/ml)	1,406	0–243
Erythrocyte sedimentation rate (mm/h)	29	< 38
Glycated-hemoglobin(%)	5.8	4.0–6.0
Alanine aminotransferase (U/L)	42	7–40
Aspartate aminotransferase (U/L)	55	13–35
γ-Glutamyl transpeptidase (U/L)	27	7–45
Alkaline phosphatase (U/L)	80	50–135
Total bilirubin (μmol/L)	4.4	3.4–20.5
Direct bilirubin (μmol/L)	2.1	0.0–6.84
Indirect bilirubin (μmol/L)	2.3	1.7–13.7
Adenosine deaminase (U/L)	42.3	4.0–22.0
Urea nitrogen (mmol/l)	4.4	2.6–7.5
Creatinine (umol/l)	74	41–73
Creatine kinase isoenzyme (ng/ml)	1.54	0–5.0
Procalcitonin(ng/ml)	0.37	0–0.05
Serum Amyloid Protein A(mg/l)	368.7	0–10.0
EBVDNA	< 5.0 × 10^3^	< 5.0 × 10^3^
CMVDNA	< 2.0 × 10^3^	< 2.0 × 10^3^
Blood plasmodium	Negative	Negative
Widal test	Negative	Negative
Hepatitis B surface antigen (IU/ml)	0.01	0.00–0.05
Hepatitis B surface antibody (mIU/ml)	18.46	0.00–10.00
Hepatitis C virus antibody	Negative	Negative
Human immunodeficiency virus antibody	Negative	Negative
Treponema pallidum antibody	Negative	Negative
Blood culture	*Brucella melitensis*	Negative

**Figure 1:**
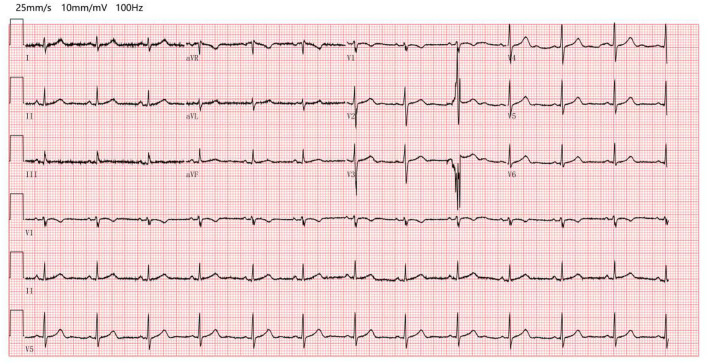
Electrocardiograms at admission (electrocardiograms showed a heart rate of only 74 beats per minute with sinus rhythm despite a body temperature of 39.1°C, without arrhythmia, no shortening of the PR segment, ST elevation or depression, and conduction block or T wave change).

**Figure 2:**
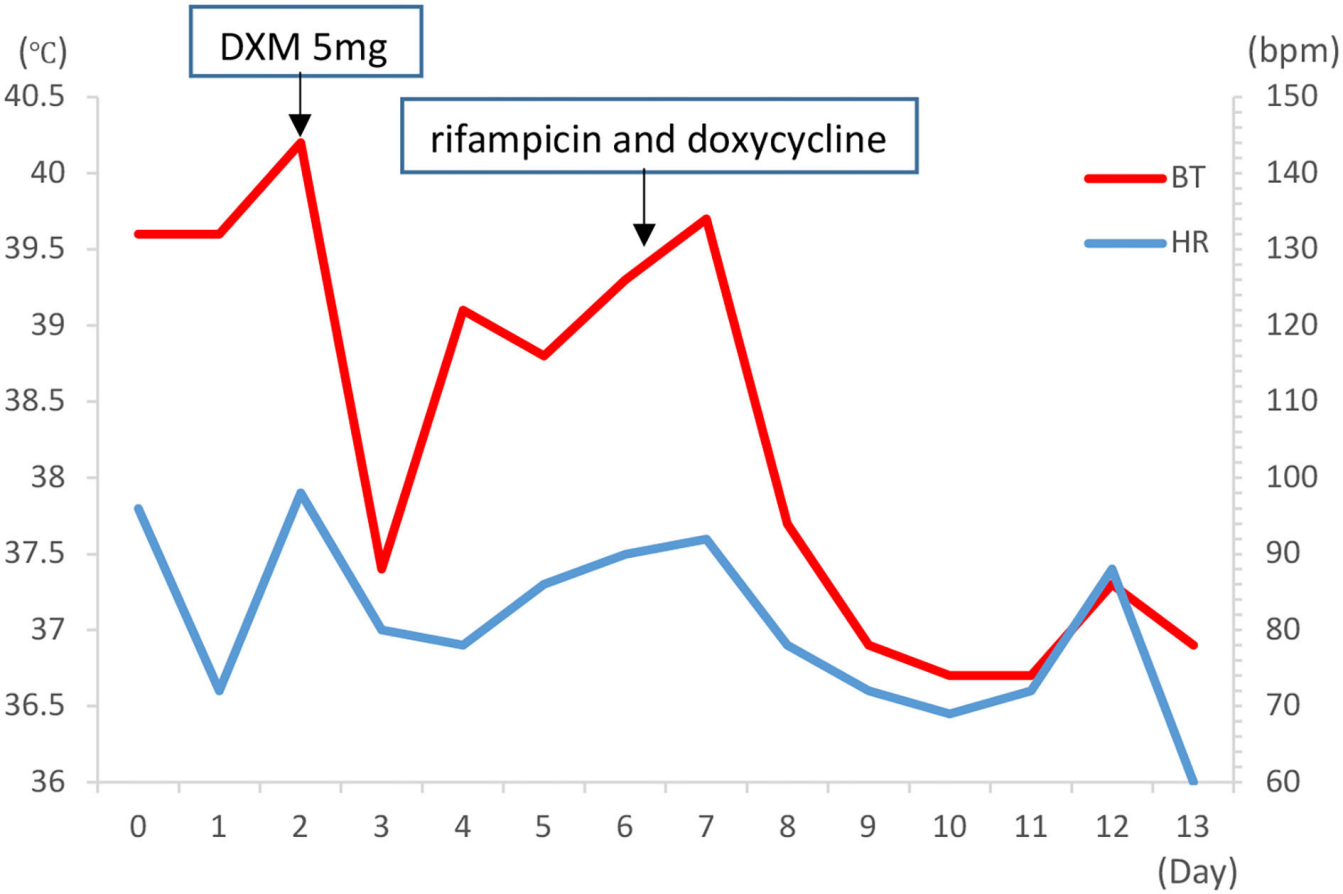
The time course of heart rate, fever, and treatment in the case (during the first week of hospitalization, persistently high fever with highest temperature >40°C, the HR remained within 70–100 bpm with sinus rhythm. bpm, beats per minute; BT, body temperature; HR, heart rate; DMX, dexamethasone).

At admission, electrocardiograms (ECGs) showed a heart rate (HR) of only 74 beats per minute (bpm) with sinus rhythm despite a body temperature of 39.1°C. The ECG demonstrated no arrhythmia, shortening of the PR segment, ST elevation or depression, conduction block, or T wave changes (Figure 1). Throughout the clinical course, the echocardiogram demonstrated normal left ventricular function and intracardiac structure, without asynergy. While hospitalization with a high fever sometimes exceeded 40°C, the HR remained between 70 and 100 bpm with a sinus rhythm (Figure 2). The patient was not receiving beta-blocker therapy. In accordance with Cunha's criteria (5), the patient was diagnosed with relative bradycardia.

On day 6, blood culture was positive for the recovery of *B. melitensis* identified by mass spectrometry (MALDI-TOF MS), and the patient was diagnosed with brucellosis. The patient resided in a part of China (Zhejiang Province) that is not considered endemic for brucellosis.

**Figure 3:**
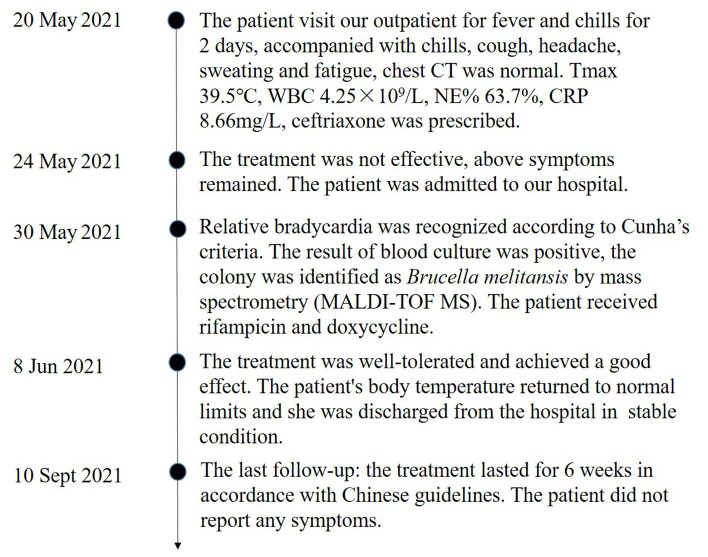
Timeline of the case.

After being diagnosed with brucellosis, the patient was treated with rifampicin (450 mg qd po) and doxycycline (100 mg bid po) for 6 weeks in accordance with Chinese guidelines (6). The patient responded to antibiotic treatment without any adverse clinical effects. After treatment, the patient's body temperature returned to normal limits on day 9 and she was discharged from the hospital in stable condition on day 13. At the latest follow-up 3 months after discharge, the patient did not report any symptoms. The whole process was concluded (Figure 3).

## Discussion

*Brucella melitensis* is the most virulent and the most prevalent worldwide among *Brucella* species. The hosts of *B. melitensis* are sheep and goats. *B. melitensis* is commonly infected by exposure to animals or animal products (1, 2). The incubation period is usually between 5 and 60 days (7). Based on clinical symptoms, epidemiological history, and positive blood culture, our case was diagnosed with brucellosis. The indications of occupational exposure had regulatory importance as the patient was from an area considered to be non-endemic for brucellosis. In the current case of *B. melitensis* infection, we observed elevated inflammatory biomarkers (CRP, PCT, and SAA) and also noted indications of damage to liver function consistent with other reports (8, 9).

Cardiac involvement (such as endocarditis, myocarditis, and pericarditis) occurs in < 2% of patients with brucellosis, but in endemic regions, this rate can increase between 7 and 10% and is the most common cause of death (10). The most common and most important complication is endocarditis (11). *Brucella* endocarditis has a predominance of aortic involvement and is prone to left ventricular failure (12). Cetin M et al. demonstrated that the parameters of ECG including Pmax, Pdis, QTmax, QTdis, QTcdis, Tp-edis interval, Tp-emax/QTmax, and Tp-emax/QTcmax ratios were significantly longer among children with brucellosis compared with healthy children, which indicates that the cardiac conduction pathways are affected (13).

Relative bradycardia is a clinical phenomenon demonstrating an insufficient increase in the heart rate despite the high fever, which may occur in some infectious diseases (including COVID-19) (14, 15). Bradycardia occurrence may correlate with the severity of the disease, as these cases were more likely to develop relative bradycardia (14), and HR tends to be lower, sometimes < 60 bpm among patients with COVID-19 (15). In the current case, HR varied from 60 to 100 bpm, and there was no evidence that relative bradycardia was associated with the severity of brucellosis.

Although mechanisms causing relative bradycardia remain unknown, inflammatory cytokines (such as interleukin 6), direct pathogenic effects on the heart (such as the sinoatrial node), increased vagal tone, and/or induction of electrolyte abnormalities have been proposed (4, 16). In the current case, cardiac biomarkers, ECG, and echocardiogram were all within normal limits and no evidence of myocardial damage was detected. The elevations in CRP, PCT and SAA suggest a role for inflammation. We considered that relative bradycardia of *B. melitensis* infection might be caused by inflammatory cytokines.

We have described and considered the potential mechanisms of a case of brucellosis with relative bradycardia. Relative bradycardia could be used as a clinical tool for the diagnosis of various disease etiologies, especially infectious diseases (4, 13). Our case demonstrates that relative bradycardia could be associated with *B. melitensis* infection, an observation that has not been previously reported. Clinicians should consider brucellosis in patients with high fever exhibiting relative bradycardia.

Some limitations were discovered in this case study. First, IL-6 was not measured, so the relation between IL-6 and relative bradycardia is not evaluated. Second, because the follow-up time was short, the long-term effect of the treatment of rifampicin and doxycycline should be assessed in future studies.

## Data availability statement

The original contributions presented in the study are included in the article/supplementary material, further inquiries can be directed to the corresponding author/s.

## Ethics statement

The study involving human participant was reviewed and approved by the Medical Ethical Committees of Ningbo First Hospital (approval number: 2022RS125). Written informed consent from the [patients/ participants OR patients/participants legal guardian/next of kin] was not required to participate in this study in accordance with the national legislation and the institutional requirements.

## Author contributions

KH and XY analyzed and interpreted the clinical data. KH, XY, YY, YC, and JS drafted the manuscript. NY and JC revised the manuscript. All authors have read and approved the manuscript.
